# Enhancement of *bla*_IMP_-carrying plasmid transfer in *Klebsiella pneumoniae* by hospital wastewater: a transcriptomic study

**DOI:** 10.3389/fmicb.2025.1626123

**Published:** 2025-07-28

**Authors:** Yuan Jiang, Luting Shu, Huan Wen, Yueshuai Wei, Siyi Liu, Caihong Ye, Ling Cheng, Zhangrui Zeng, Jinbo Liu

**Affiliations:** ^1^Department of Laboratory Medicine, The Affiliated Hospital of Southwest Medical University, Sichuan Province Engineering Technology Research Center of Molecular Diagnosis of Clinical Diseases, Molecular Diagnosis of Clinical Diseases Key Laboratory of Luzhou, Luzhou, China; ^2^People’s Hospital of Xuyong County, Luzhou, China; ^3^Hospital-Acquired Infection Control Department, The Affiliated Hospital of Southwest Medical University, Luzhou, China

**Keywords:** hospital wastewater, HGT, ARGS, conjugation, *Klebsiella pneumoniae*

## Abstract

**Introduction:**

*Klebsiella pneumoniae* is a critical ESKAPE pathogen that presents a significant challenge to public health because of its multidrug-resistant strains. This study investigates the impact and mechanisms of hospital wastewater on the horizontal gene transfer of carbapenem resistance genes, particularly *bla*_IMP_, in *K. pneumoniae*.

**Methods:**

LB broth was prepared using sterile filtered wastewater as the substrate to investigate the impact of wastewater on the transfer of carbapenem-resistant gene *bla*_IMP_ in *K. pneumoniae*. The mechanisms of sewage effects on the horizontal transfer of *bla*_IMP_ were explored by integrating transcriptome sequencing with the detection of extracellular membrane permeability, intracellular reactive oxygen species (ROS), and other test results.

**Results:**

Hospital wastewater significantly enhances the conjugation frequency of plasmids containing *bla*_IMP_, showing a two-fold increase in wastewater-based LB broth compared to regular LB broth. In comparison to regular LB broth culture, the wastewater-based LB broth culture group showed significant alterations in the expression of 1,415 genes, with 907 genes upregulated and 508 genes downregulated. Genes related to conjugation transfer systems and the type IV secretion system were significantly upregulated, indicating a potential role in promoting plasmid transfer. Moreover, the treatment of wastewater resulted in elevated intracellular ROS production and increased permeability of bacterial outer membranes, potentially facilitating the spread of antibiotic resistance genes.

**Discussion:**

This research shows that hospital wastewater facilitates the transfer of drug-resistant plasmids containing *bla*_IMP_ and elucidates its potential mechanisms. A more detailed investigation into these mechanisms may facilitate the prevention of resistance transmission between healthcare and environmental contexts and inform future strategies for managing carbapenem resistance.

## 1 Introduction

*Klebsiella pneumoniae* is a part of the ESKAPE pathogens group, which also include *Enterococcus* spp., *Staphylococcus aureus*, *Acinetobacter baumannii*, *Pseudomonas aeruginosa*, and *Enterobacter* spp. This bacterium is known to cause both community-acquired and hospital-acquired infections, such as pneumonia, urinary tract infections, hepatobiliary infections, and sepsis (Martin and Bachman, 2018). In recent years, there has been an increase in the detection of multidrug-resistant *K. pneumoniae* (Jalal et al., 2023). Of the estimated 4.95 million deaths related to bacterial antimicrobial resistance in 2019, 1.27 million were directly connected to the disease (Antimicrobial Resistance Collaborators, 2022). Due to the limited options for antibiotic treatment and the high mortality rate linked to multidrug-resistant *Klebsiella pneumoniae*, there is a serious threat to human health on a global scale (Holt et al., 2015).

Carbapenems were once seen as a last resort for treating multidrug-resistant Gram-negative bacterial infections in critically ill patients. However, the widespread use of carbapenems has led to the emergence and spread of carbapenem-resistant *K. pneumoniae* (CRKP) on a global scale, presenting new challenges to public health security (Navon-Venezia et al., 2017; Cassini et al., 2019). Carbapenemases are the most common mechanism of resistance to carbapenem antibiotics in bacteria. They can be classified into Class A serine carbapenemases, Class B metallo-β-lactamases, and Class D OXA-48-type serine carbapenemases according to the Ambler classification (Queenan and Bush, 2007; Jeon et al., 2015). KPC, which is encoded by *bla*_KPC_, is the most common carbapenemase in Class A and the primary resistance mechanism of CRKP to carbapenem antibiotics. NDM, IMP, and VIM are the representative class B metallo-β-lactamases. In China, the KPC-type enzyme is the predominant carbapenemase, while the NDM-type enzyme comes in second and the detection rate of IMP-type enzyme is relatively low (Zhang et al., 2018).

In recent years, there has been an increase in the detection rates of metal-lo-β-lactamases such as NDM and IMP in *K. pneumoniae* due to the widespread transfer of antibiotic resistance genes between bacteria (Kubota et al., 2019; Xu J. et al., 2020). However, *K. pneumoniae* carrying *bla*_IMP_ results in lower levels of carbapenem resistance, which can be easily overlooked, leading to underestimation of the presence of IMP in CRKP (Chen et al., 2009). Bacteria can develop resistance through horizontal gene transfer (HGT) due to various external factors, allowing for the evolution of resistance. Plasmid-mediated conjugative transfer is considered the main method of horizontal transfer of antibiotic resistance genes (ARGs) between bacteria (San Millan, 2018; Lerminiaux and Cameron, 2019). Studying the mechanisms of plasmid-mediated conjugative transfer will help to develop corresponding intervention measures to curb the horizontal transfer of ARGs between bacteria.

Researchers have increasingly recognized the role of the environment in the spread of bacterial resistance in recent years. High levels of microbial pathogens, antibiotics and related metabolites, metals, and resistant bacteria that may carry ARGs in hospital wastewater collectively form a complex environmental reservoir of bacteria (Katagiri et al., 2021; Svebrant et al., 2021). The complex microbial environment and high genetic diversity facilitate the horizontal transfer of ARGs between bacteria through HGT (Zhang K. et al., 2021). An increasing number of researchers have been investigating the effects of various pollutants in the environment, such as biocides residues (Zhang et al., 2023), paclitaxel and its derivatives (Yang et al., 2022), phthalates (Wu et al., 2023), heavy metal ions (Palm et al., 2022), and residual disinfectants (He et al., 2022), on HGT. It has been found that these pollutants can promote the horizontal spread of resistance genes in the environment, with changes in cell membrane permeability, oxidative stress, and the expression of genes related to conjugation playing critical roles. However, most of these studies focus on specific components of pollutants, and there are few reports using real-world wastewater to study gene-level transfer.

Higher concentrations of organic matter, microplastics, and antibiotics in sewage have been shown to interact with a large number of microorganisms, which in turn promotes the production and spread of ARGs (Zhao et al., 2024). Brown et al. explored the impact of sewage-related antibiotics on the development of antibiotic resistance, and their findings supported the idea that changing levels of antibiotics in sewage are linked to horizontal gene transfer of ARGs (Brown et al., 2024). Recently, Basiry et al. (2025) discovered that antibiotics at sub-minimum inhibitory concentrations enhanced the spread of ARGs among the bacterial community in activated sludge sequencing batch reactors in the laboratory. Studying HGT through actual sewage has emerged as a popular research area in the transmission of ARGs. This research utilizes transcriptomic sequencing, along with outer membrane permeability testing and intracellular reactive oxygen species detection in bacteria, to investigate the potential mechanisms of horizontal transfer of the carbapenem resistance gene *bla*_IMP_ facilitated by wastewater in *K. pneumoniae*. The goal is to establish a theoretical foundation for preventing the dissemination of resistant bacteria and resistance genes in wastewater, as well as to provide a scientific basis for managing the continued progression of carbapenem antibiotic resistance.

## 2 Materials and methods

### 2.1 The collection and treatment of hospital sewage

Sewage samples were collected from the influent of a sewage treatment plant associated with an affiliated hospital of medical university. Once collected, the sewage samples were centrifuged at 4°C and 4,000 r/min for 10 min. Filtered the supernatant through a disposable sterile filter with a pore size of 0.22 μm, repeating the filtration process three times. The filtered samples were stored in a 4°C refrigerator for future use. The chemical composition of sewage was detected through non-targeted analysis by Hangzhou Yanqu Information Technology Co., Ltd. (Hangzhou, China), using Liquid Chromatograph Mass Spectrometer.

### 2.2 Culture media and antibiotics

LB broth and LB agar were purchased from Guangdong Huankai Microbial Science and Technology Co., Ltd. (Guangzhou, China). Rifampicin was sourced from Heowns Biotechnology Co., Ltd (Tianjin, China), and meropenem was obtained from Shanghai Macklin Biochemical Technology Co., Ltd. (Shanghai, China). LB broth was prepared by dissolving 36 g of LB broth dry powder in 1 L of deionized water. LB broth consists of 10 g Peptone, 5 g NaCl, 5 g yeast extract, and 1 g dextrose per 1 L; LB agar includes 15 g agar per 1 L of LB broth. The mixture was heated, agitated frequently, and boiled until the powder was completely dissolved. Subsequently, the medium was sterilized in an autoclave at a temperature of 121°C for 15 min. To prepare wastewater-based LB broth, the supernatant of wastewater was used to instead of deionized water. 1,000 mL of wastewater supernatant was measured using a sterilized screw-cap bottle and pressure cooker. 36 g LB broth dry powder was then weighed and added to the measured wastewater supernatant in the bottle. The bottle was shaken until the powder completely dissolved, resulting in clear and transparent broth. The prepared wastewater-based LB broth was filtered three times using a disposable sterile filter with a pore size of 0.22 μm. The filtered broth was then transferred to a sterile container and stored in a refrigerator at 4°C until ready for use.

### 2.3 Donor and recipient strains

Utilizing a clinically sourced strain of carbapenem-resistant *K. pneumoniae* as the donor strain, which harbors an IncN_1 type plasmid containing *bla*_IMP_ and *qnrS1*, as well as a plethora of genes linked to conjugation and the type IV secretion system (T4SS) (Supplementary Figure S1). Plasmids belonging to the IncN group are typically known for their wide host range and efficient transmission, making them crucial for the spread of key resistance genes (Feng et al., 2016; Chi et al., 2020). The recipient strain for the experiment was *Escherichia coli 600* (rifampicin-resistant) maintained by our laboratory.

### 2.4 Conjugative transfer experiments

Both the donor and recipient bacteria were inoculated onto Columbia blood agar plates and allowed to culture overnight at 35°C in an incubator. A single fresh colony on Columbia blood agar plates was selected and inoculated into 5 mL of broth. The donor bacteria were cultured in LB broth and wastewater-based LB broth separately, while the recipient bacteria were cultured in LB broth. The cultures were then incubated at 35°C with shaking at 160 r/min for 18–24 h. After incubation, the broth was centrifuged, and the supernatant was removed. The bacterial concentration was adjusted using LB broth to prepare a bacterial suspension. 200 μL of the donor bacterial suspension was mixed with 600 μL of the recipient bacterial suspension in a 1.5 mL sterile EP tube, and the mixture was vortexed to ensure thorough mixing. The mixture was then placed in a 35°C incubator for static culture for 4 h.

After incubation, 100 μL of the conjugation mixture was inoculated on selective LB agar plates containing marker antibiotics (meropenem 2 μg/mL and rifampicin 500 μg/mL). Plates were incubated at 35°C for 24 h. The presumed conjugants were purified and then validated by MALDI-TOF MS and colony PCR to confirm the successful plasmid conjugation. Primer sequences and amplification condition are shown in Supplementary text S1. The colonies of transconjugants and recipients were then counted, respectively. All treatments were carried out in triplicate. The conjugative transfer frequency is determined by the ratio of transconjugants (CFU/mL) to recipients (CFU/mL).

### 2.5 Determination of reactive oxygen species and cell membrane permeability

To understand the underlying mechanisms of improved conjugation, it is crucial to explore whether the elevated generation of bacterial reactive oxygen species (ROS) and the subsequent rise in cell membrane permeability act as the main driving factor. The levels of intracellular ROS were assessed by utilizing the ROS assay kit (Biyuntian, China), following the guidelines provided by the manufacturer. Cell membrane permeability was indicated by the fluorescence intensity of propidium iodide (PI) and 1-N-phenylnaphthylamine (NPN) as described in previous studies (Elliott et al., 2020; Yu et al., 2021).

### 2.6 Transcriptome sequencing and bioinformatic analysis

RNA-seq was conducted to analyze the transcriptional expression levels in the donor under various culture conditions, including sewage matrix LB broth and normal LB broth. Fresh donor bacterial colonies were handpicked from the blood agar plates and introduced into 50 mL of wastewater-based LB broth and standard LB broth. After incubating the samples at 37°C for 16 h on a constant temperature shaker, they were centrifuged at 8,000 r/min for 10 min to harvest the bacterial cells. The sediment was then placed into a sterile 1.5 mL cryovial, rapidly frozen in liquid nitrogen for 5–10 min, and subsequently stored at –80°C for temporary preservation. RNA isolation, rRNA removal, strand-specific cDNA library construction, and sequencing were conducted by Shanghai Major Biomedical Technology Co., Ltd. (Shanghai, China). Transcriptomic bioinformatics analysis and visualization were conducted based on the MajorBio Cloud platform^1^ and R language. The specific sequencing process, methods, and bioinformatics analysis are detailed in Supplementary text S2.

### 2.7 Statistical analysis

The Student’s *t*-test was employed for statistical analysis of the conjugation frequency, outer membrane permeability, and ROS production. A *P-*value of < 0.05 was considered statistically significant. GraphPad 8.0 was used for data visualization.

## 3 Results

### 3.1 Hospital sewage facilitates the horizontal transfer of *bla*_IMP_

**FIGURE 1:**
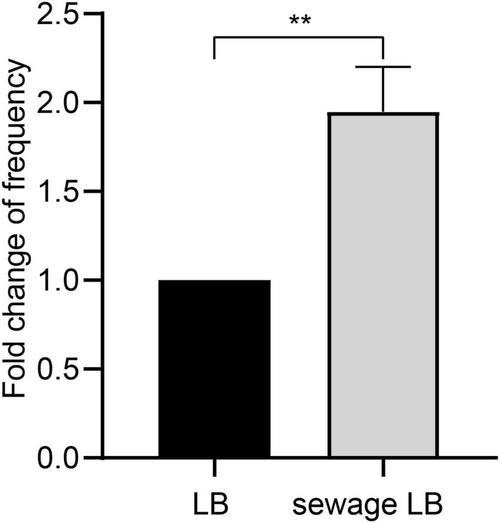
Differences of conjugation frequency after different broth cultures. LB, Normal LB broth culture; Sewage LB, Sewage substrate LB broth culture; **P < 0.01.

Non-targeted chemical composition analysis reveals the presence of a rich variety of organic pollutants in the hospital sewage (Supplementary Table S1). Among these pollutant components, Metformin (Guo et al., 2024) and Acesulfame (Yu et al., 2021) are organic pollutants that have been shown to enhance the dissemination of antibiotic resistance through horizontal conjugative gene transfer. In order to examine how real wastewater affects the horizontal transfer of the carbapenem resistance gene *bla*_IMP_, the donor strain was initially cultured in LB broth based on wastewater. The changes in conjugation frequency were then observed through a conjugation experiment. The results showed that after culturing in wastewater-based LB broth, the conjugation frequency was approximately twice that of normal LB broth culturing, with a statistically significant difference (*P* < 0.05, Figure 1). This indicates that wastewater can promote the horizontal spread of ARGs by enhancing the conjugation transfer of plasmids between bacteria.

### 3.2 Influence of hospital sewage on bacterial gene expression profiles

**FIGURE 2:**
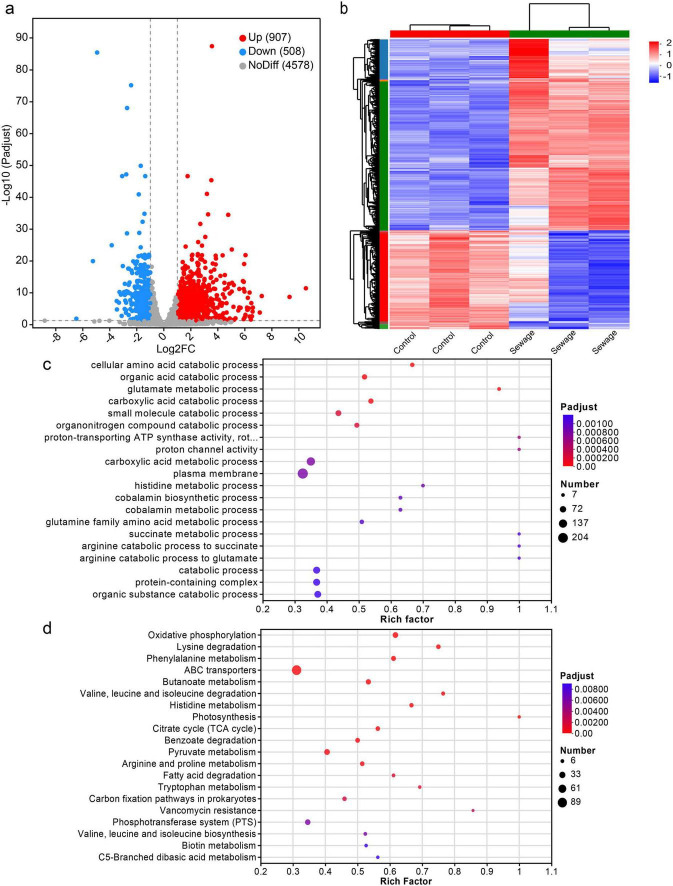
Differentially expressed genes and their functional enrichment analysis. **(A)** Volcano plot of differential expression. **(B)** Clustering heatmap of all DEGs. **(C)** Bubble plot of GO enrichment analysis of DEGs. **(D)** Bubble plot of KEGG enrichment analysis of DEGs.

As shown in Figure 1, Sewage substrate LB broth can promote the conjugation transfer between bacteria. Therefore, to investigate the impact of wastewater on conjugation transfer, strains JYKP3 were cultured in both wastewater-based LB broth and normal LB broth for transcriptomic sequencing. DESeq2 was used to identify differentially expressed genes (DEGs) based on the criteria of |log2 FC| > 1 and *P*_*adjust*_ < 0.05. The result showed that compared to normal LB broth culture, there were 1,415 genes with significantly altered expression in the wastewater-based LB broth culture group, with 907 genes upregulated and 508 genes downregulated (Figure 2A). Cluster analysis indicated a clear clustering relationship between the two groups of DEGs (Figure 2B). It is suggested that culturing in wastewater-based LB broth results in a distinct gene expression profile in bacteria, potentially facilitating the transfer of plasmids through conjugation.

### 3.3 GO and KEGG enrichment analysis of DEGs

In order to annotate the biological functions of the identified DEGs, Gene Ontology (GO) and Kyoto Encyclopedia of Genes and Genomes (KEGG) enrichment analyses were conducted on these genes individually. The top 20 GO enrichment results showcasing the varying degrees of enrichment of identified DEGs in biological processes, molecular functions, and cellular components under the condition of *P*_*adjust*_ < 0.05 (Figure 2C). The cellular components mainly consist of plasma membranes and protein complexes, while the molecular functions primarily involve the activity of proton transporter ATP synthase and proton channel activity. The biological processes include cellular amino acid catabolism, organic acid metabolism, glutamic acid metabolism, carboxylate metabolism, and small molecule catabolism. Notably, the plasma membrane and carboxylate metabolism processes have the highest number of enriched differentially expressed genes, with 204 and 132 DEGs, respectively.

The results of top 20 KEGG enrichment in the condition of *P*_*adjust*_ < 0.05 indicate that the identified DEGs are predominantly enriched in ABC transporters, diverse metabolic pathways, and the production of secondary metabolites (Figure 2D). Of these, ABC transporters show the highest enrichment, with 95 DEGs, while pyruvate metabolism follows with 33 DEGs enriched.

### 3.4 Differential expression of conjugation-related genes after hospital sewage treatment

**FIGURE 3:**
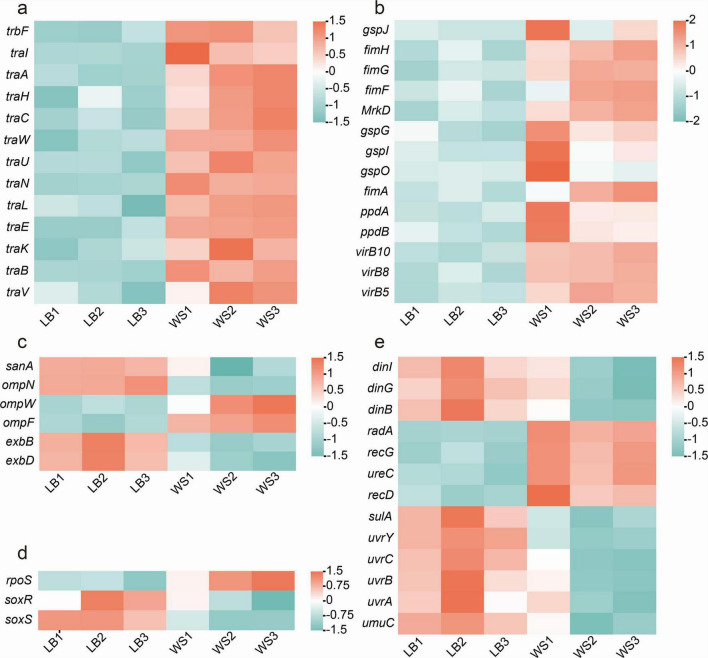
DEGs related to conjugation. **(A)** Differentially expressed conjugation-related genes; **(B)** DEGs related to type IV secretion system and pili production; **(C)** DEGs of extracellular membrane proteins; **(D)** differential expression of ROS-related genes; **(E)** differential expression of SOS-related genes.

Based on the initial sequencing data, the genome of donor strain is found to have numerous genes linked to conjugative transfer. The expression levels of genes associated with conjugative transfer were analyzed among the DEGs identified. The results revealed that genes responsible for the conjugative transfer system, such as *traA*, *traB*, *traC*, *traK*, *traH*, *traF*, *traE*, *traW*, *traU*, *traN*, *traL*, and *traV*, were highly expressed in the experimental group (Figure 3A). Additionally, genes encoding the Type IV secretion system, including *virB5*, *virB8*, and *virB10*, as well as flagellum production-related genes *gspJ*, *gspG*, *gspL*, *gspO*, *fimH*, *fimG*, *fimF*, *fimA*, *ppdA*, and *ppdB*, showed significant upregulation in the experimental group (Figure 3B).

Nevertheless, genes involved in ROS production and SOS response regulation, along with genes influencing bacterial outer membrane permeability, exhibit distinct expression patterns in the experimental group compared to the control group. The results demonstrate that in the control group, genes encoding outer membrane proteins such as *ompN*, *exbB*, and *exbD* are highly expressed, whereas in the experimental group, *ompW* and *ompF* exhibit high levels of expression (Figure 3C). The gene *rpoS*, which is associated with oxidative stress, is highly expressed in the experimental group, while the genes *soxS* and *soxR* are expressed in the control group (Figure 3D). Additionally, genes involved in regulating the SOS response, such as *dinI*, *dinG*, *dinB*, *radA*, *uvrY*, *uvrC*, *uvrA*, *uvrB*, and *umuC*, are downregulated in the experimental group, while *recD*, *recG*, *sulA*, and *ureC* are significantly upregulated in the experimental group (Figure 3E). The different expression patterns of these genes with similar functions may be linked to changes in the metabolic state of bacteria in the wastewater matrix LB broth, as indicated by the enrichment of differentially expressed genes in metabolic and secondary metabolite biosynthesis processes.

### 3.5 Hospital sewage leads to an increase in the permeability of bacterial outer membranes

**FIGURE 4:**
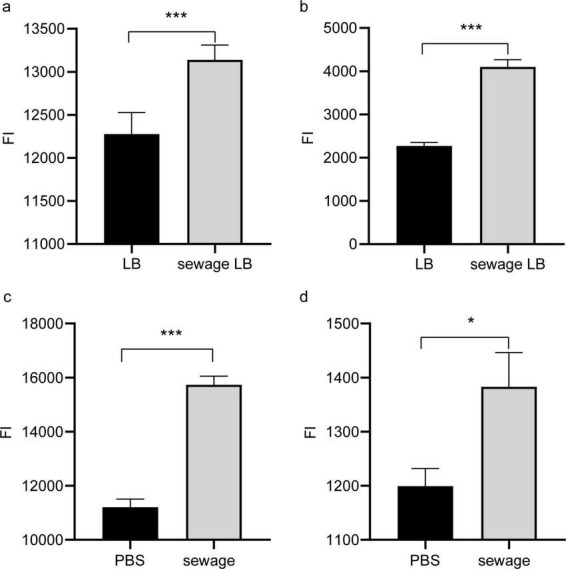
Permeability of outer membrane of bacterial. **(A)** NPN detection of the effect of LB broth culture on membrane permeability in wastewater substrate; **(B)** PI detection of the effect of LB broth culture on membrane permeability in wastewater substrate; **(C)** NPN detection of the impact of direct treatment of wastewater on bacterial membrane permeability; **(D)** PI detection of the impact of direct treatment of wastewater on bacterial membrane permeability. LB, Normal LB broth culture; Sewage LB, Sewage substrate LB broth culture; *P < 0.05, ***P < 0.001.

To investigate the role of outer membrane permeability in promoting conjugative transfer of plasmids in wastewater, this study used NPN and PI analysis to assess changes in bacterial outer membrane permeability after culture in LB broth with wastewater matrix and direct wastewater treatment. Both assays revealed a notable rise in bacterial outer membrane permeability following culture in LB broth with the wastewater matrix and undergoing direct wastewater treatment, with a statistical significance (*P* < 0.05, Figure 4). The enhanced permeability of the outer membrane in bacteria allowed for easier conjugative transfer of plasmids between them, ultimately aiding in the horizontal dissemination of antibiotic resistance genes during the wastewater treatment process.

### 3.6 Effect of hospital sewage on the production of intracellular ROS in bacteria

The accumulation of intracellular ROS is considered to be one of the key mechanisms by which environmental organic pollutants impact bacterial conjugative transfer. Studies conducted previously have revealed that hospital wastewater samples are filled with various organic pollutants like drug residues, drug metabolites, and organic solvents (Verlicchi et al., 2015). The results of transcriptome sequencing revealed distinct expression patterns of genes associated with oxidative stress in the experimental group. As a result, this study further investigates the mechanisms associated with plasmid conjugative transfer facilitated by wastewater by observing changes in intracellular ROS levels after incubation in LB broth supplemented with a wastewater matrix and direct exposure to wastewater.

**FIGURE 5:**
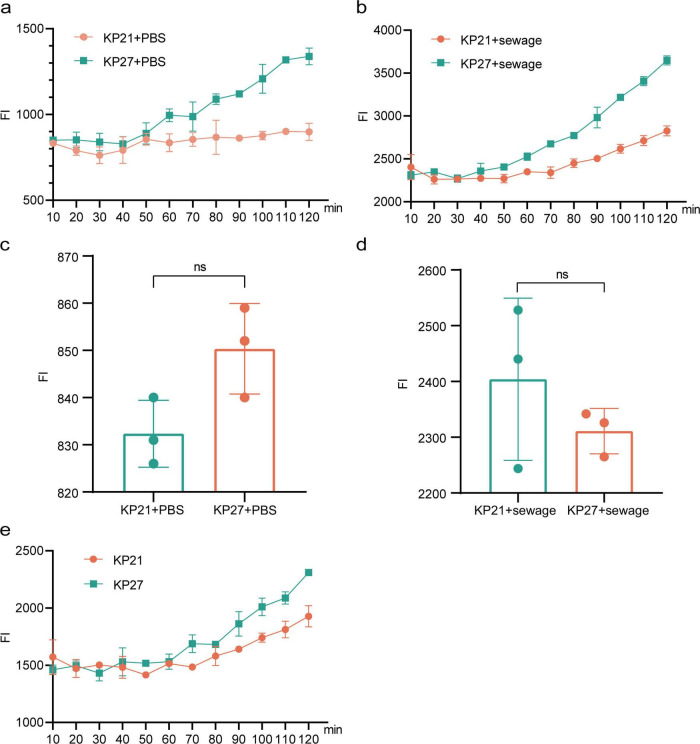
The effect of different processing methods on bacterial ROS. **(A)** Changes in ROS over time after adding PBS to KP21 and KP27; **(B)** Changes in ROS over time after adding wastewater to KP21 and KP27; **(C)** ROS detection results after 10 min of adding PBS to KP21 and KP27; **(D)** ROS detection results after 10 min of adding sewage to KP21 and KP27; **(E)** The ROS detection results of wastewater treatment minus PBS treatment change over time. KP21, Normal LB broth passage; KP27, Sewage substrate LB broth passage; ns, no significant difference.

Initially, bacterial passage culture was carried out using regular LB broth and LB broth derived from wastewater, which led to the isolation of strain KP21 (cultured in regular LB broth) and strain KP27 (cultured in wastewater-based LB broth). Following treatment with wastewater and PBS, the alterations in intracellular ROS levels between the two strains, KP21 and KP27, were compared. The results indicate that while there was no notable disparity in intracellular ROS levels after 10 min of treatment, a significant difference emerged as the treatment duration extended (Figures 5A,B). Specifically, in the PBS treatment group (Figures 5A,C), there was no significant change in intracellular ROS levels over time in KP21; however, in KP27, intracellular ROS levels showed a gradual increase. The wastewater treatment group (Figures 5B,D) displayed a considerably higher increase in intracellular ROS in KP27 compared to KP21. This indicates that strains passaged in wastewater-based LB broth continue to exhibit higher levels of intracellular ROS even after the removal of external stimuli. Moreover, the strain that underwent passages in LB broth derived from wastewater exhibited a heightened response to external oxidative triggers, as evidenced by the difference in ROS levels between the wastewater treatment and PBS treatment groups (Figure 5E).

**FIGURE 6:**
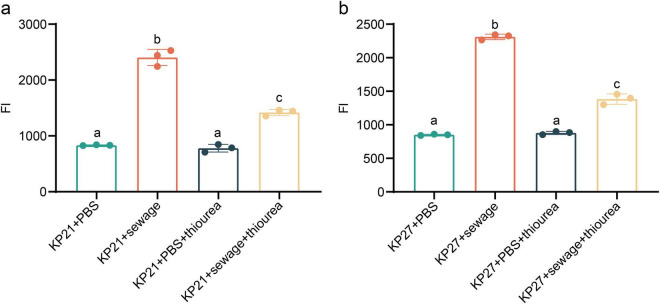
The effect of sewage on bacterial ROS. **(a)** The effect of sewage on ROS of bacteria passaged by normal LB broth. **(b)** The effect of sewage on ROS of bacteria passaged by sewage-based LB broth. KP21: Normal LB broth passage; KP27: Sewage-based LB broth passage. The final concentration of thiourea is 100 mmol/L. The difference between different lowercase letters is statistically significant (*P* < 0.05).

**FIGURE 7:**
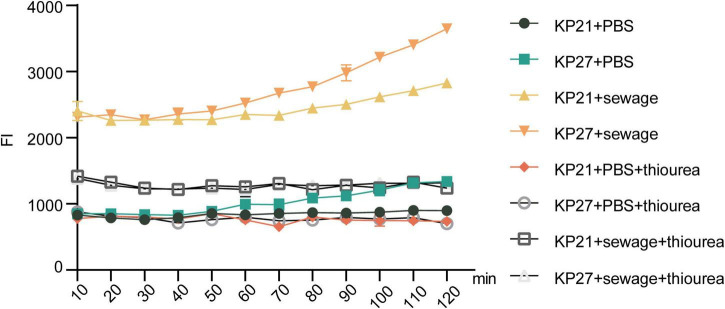
Results of bacterial ROS after passage in different broth. KP21, Normal LB broth passage; KP27, Sewage substrate LB broth passage. The final concentration of thiourea is 100 mmol/L.

Upon the addition of thiourea, a strong reducing agent, to the experimental system, it was observed that there was no significant alteration in ROS levels in either KP21 or KP27 in the PBS treatment group (*P* > 0.05, Figure 6). Nevertheless, in the wastewater treatment group, thiourea managed to lower the ROS levels triggered by wastewater stimulation, albeit a notable difference persisted compared to the PBS treatment group (*P* < 0.05, Figure 6). The results of ROS detection in bacteria (KP21, KP27) after being cultured in various broths and subjected to different experimental conditions are summarized in Figure 7. Following the addition of thiourea, there was no change in intracellular ROS levels over time in both the PBS and wastewater treatment groups. Nevertheless, the intracellular ROS levels in the wastewater treatment group were consistently higher than those in the PBS treatment group.

## 4 Discussion

Horizontal transfer of ARGs between bacteria plays a crucial role in the swift advancement of bacterial antibiotic resistance. The “One Health” concept has expanded the spread of ARGs beyond hospital settings, establishing an interconnected transmission network involving humans, animals, and the environment (Kim and Cha, 2021; Zhuang et al., 2021). Monitoring data from “One Health” reveals that the abundance of ARGs in hospital wastewater surpasses that in domestic wastewater by 2–3 orders of magnitude, while showing a homology of over 89% with clinical isolates (Cai et al., 2021). Within the hospital sewage system, there is a unique microenvironment that contains sub therapeutic levels of antibiotics, heavy metal pollutants, and drug-resistant strains, making it an ideal breeding ground for the evolution and dissemination of ARGs (Hassoun-Kheir et al., 2020).

Following the findings by Qiu et al. (2012) regarding the ability of nano-sized aluminum oxide particles in wastewater to facilitate the horizontal transfer of plasmid-mediated ARGs, researchers are now exploring the impact of various pollutants that could be present in wastewater on the transfer of ARGs. Wastewater is a complex integrated system, consisting of a high level of microbial pathogens, drugs and related metabolites, heavy metal ions, and other pollutants, collectively forming a complex environmental reservoir of bacteria (Katagiri et al., 2021; Svebrant et al., 2021). The final biological response in wastewater is frequently the outcome of a blend of various substances. However, it is still unknown whether this combined effect is in line with results from laboratory research. Due to the limitations of experimental conditions and techniques, the majority of studies tend to concentrate on just one or a few pollutant constituents in wastewater, with only a small number of reports utilizing simulated sewage to investigate the horizontal transfer of drug resistance genes.

In this study, wastewater-based LB broth was prepared based on the sterile filtrate of wastewater from the influents of a hospital wastewater treatment plant, and it was found that wastewater-based LB broth significantly enhanced the horizontal transfer of a drug-resistant plasmid containing *bla*_IMP–4_ compared to regular LB broth culture. Transcriptomic sequencing analysis showed distinct gene expression patterns in the two broth culture conditions, with 907 genes upregulated and 508 genes downregulated. The differentially expressed genes were predominantly enriched in biological pathways related to the formation of plasma membrane, metabolism of carboxylic acid, ABC transporters, and pyruvate metabolism.

The mating pair formation system and DNA transfer and replication system play a crucial role in the regulation of plasmid conjugative transfer (Miyakoshi et al., 2020). It has been found that exogenous stress or stimulation can affect the frequency of plasmid conjugative transfer by altering the expression of these related genes (Xu L. et al., 2020; Zhang H. et al., 2021). The expression of genes related to the conjugation transfer system in donor bacteria, including *traA*, *traB*, *traC*, *traK*, *traH*, *traF*, *traE*, *traW*, traU, *traN*, *traL*, and *traV*, were significantly upregulated after cultivation in the wastewater-based LB broth. Despite the fact that these genes are not all identified on the plasmid containing *bla*_IMP–4_ according to the sequencing information, researches have shown that the co-integration of plasmids plays a crucial role in bacterial genetic diversity and the horizontal transmission of ARGs (Li et al., 2020; Liu et al., 2021). Although some R plasmids do not contain genes for conjugation transfer, they can still overcome transmission barriers by working with conjugation plasmids to facilitate the horizontal transfer of ARGs (Li et al., 2020). Thus, the increased expression of these genes strengthens the conjugation system, which is essential for the transfer of drug-resistant genes. In summary, hospital sewage has the potential to enhance the conjugation transfer of plasmids containing *bla*_IMP–4_ by influencing the expression of genes involved in conjugation transfer, like *traB*, *traC*, and *traK*.

The T4SS is a multifunctional transport system found in bacteria that plays a crucial role in transferring ARGs between bacteria (Ilangovan et al., 2015; Bergé et al., 2017). It not only transports cellular effector proteins and toxins but also facilitates HGT by either conjugative transfer or delivering genetic material to recipient cells. The T4SS found in Gram-negative bacteria consists of 12 structural proteins, specifically VirB1 to VirB11 and VirD4 (Bergé et al., 2017; Waksman, 2019). According to the transcriptomic analysis conducted in this study, the wastewater-based LB broth culture group exhibited a significant upregulation in the expression of *virB5*, *virB8*, and *virB10*. Despite the fact that not all T4SS encoding genes experienced an upregulation, the significantly upregulated *virB10* plays a key role in the outer membrane core complex of T4SS. Its main function is to form an outer membrane channel that connects the inner and outer membranes of bacteria (Costa et al., 2021). Consequently, these T4SS structural genes that are expressed differently may be key players in enhancing plasmid conjugative transfer in wastewater.

Additionally, a large number of flagellum-related genes were found to be upregulated in the wastewater-based LB broth culture group through transcriptomic sequencing. In the investigation of how environmental fungicides affect the transfer of drug-resistant plasmids, Zhang et al. (2023) observed that flagellum-related genes like *fimA*, *ppdA*, and *ppdB* were upregulated to varying degrees and might have a role in plasmid conjugation transfer, which is in line with our research outcomes. While not all flagella are associated with conjugative transfer, the presence of additional flagella can lead to bacteria aggregating or forming biofilms on surfaces. According to the latest study led by Element et al. (2023), it has been discovered that the growth of biofilms significantly elevates the risk of conjugative transfer of carbapenem resistance plasmids in *K. pneumoniae*. This occurs without the necessity for adaptive costs or changes in transcription, highlighting the importance of biofilms in the propagation of ARGs. Therefore, these flagellum-related genes that are expressed at high levels could potentially boost plasmid conjugative transfer by facilitating bacterial cell aggregation or biofilm formation.

The successful transfer of plasmids between bacteria is dependent on various physiological and biochemical conditions. Factors like intracellular ROS production, the SOS response, and outer membrane permeability all contribute to the efficiency of plasmid conjugative transfer (Wang et al., 2019). A variety of studies have shown that genes with similar functions do not always exhibit the same expression patterns under identical conditions (Zhou et al., 2021; Yu et al., 2023; Guo et al., 2024). In the same vein, our findings also revealed that genes with similar functions exhibited varying expression profiles within the experimental group. ROS can directly target bacterial DNA, proteins, or cell membranes, leading to cell damage, altering gene and protein expression, and ultimately impacting the transfer of ARGs among bacteria (Winterbourn, 2008; Li and Zhang, 2022). Even though the analysis of differential gene expression revealed an increase in *rpoS* expression and a significant decrease in *soxS* and *soxR* expression following treatment with wastewater-based LB broth, the monitoring of intracellular ROS production and its temporal changes, along with the trend of intracellular ROS levels after the addition of the potent reducing agent thiourea, demonstrated a substantial enhancement in intracellular ROS production due to the sewage treatment.

When environmental factors directly target the cell membrane or induce an elevation in intra-bacterial ROS levels, these ROS can in turn damage the cell membrane. This damage increases the permeability of the bacterial cell membrane, allowing for the easier transfer of ARGs across the membrane and thereby promoting the horizontal transfer of ARGs between bacteria (Li and Zhang, 2022). Studies by Zhang et al. (2023) have found that after exposure to biocides, bacterial outer membrane permeability increases, and genes encoding outer membrane proteins such as ompA, ompC, and ompF are upregulated. Similar conclusions were also drawn by He et al. (2022) based on research on disinfectant residues. During this investigation, there was a notable increase in outer membrane permeability observed when culture in wastewater-based LB broth and direct treatment of bacteria with wastewater, as detected by NPN and PI assays. However, the expression of outer membrane protein-encoding genes *ompN* was downregulated in the experimental group, which may be related to the metabolic state of the bacteria in wastewater. Even though *ompN* expression was downregulated, outer membrane permeability still increased, potentially because of the elevated expression of outer membrane permeability proteins like *ompW* and *ompF*. Additionally, the SOS response may also alter cell membrane permeability and DNA expression (Li and Zhang, 2022). It is hypothesized that sewage may cause direct damage to cell membranes or indirectly lead to increased membrane permeability by triggering SOS response and the production of ROS, ultimately facilitating the transfer of ARGs through plasmids.

In general, our research confirms the potential role of hospital wastewater in the transmission of drug resistance in humans, animals, and the environment. This offers a fresh perspective on managing bacterial resistance within the framework of “one health.” Nevertheless, it is important to acknowledge the limitations of this study. Variations in the composition of pollutants in hospital wastewater across different regions and seasons could result in significant differences in the transfer of ARGs. Moreover, further research is needed to explore the roles and interactions of multiple pollutant components in wastewater in relation to the HGT of drug-resistant genes.

## 5 Conclusion

In conclusion, hospital wastewater plays a role in the spread of resistance plasmids containing *bla*_IMP–4_ by influencing gene expression linked to conjugation, boosting the secretion systems and pili production, and increasing ROS production and bacterial outer membrane permeability. Studying these mechanisms further can aid in preventing resistance transmission between healthcare and environmental contexts, and guide efforts to manage carbapenem resistance moving forward.

## Data Availability

The original datasets presented in the study are publicly available. This data can be found here: https://www.ncbi.nlm.nih.gov/, PRJNA1293672.

## Footnotes

1
https://cloud.majorbio.com

## References

[B1] Antimicrobial Resistance Collaborators (2022). Global burden of bacterial antimicrobial resistance in 2019: A systematic analysis. *Lancet (London, England)* 399 629–655. 10.1016/S0140-6736(21)02724-0 35065702 PMC8841637

[B2] Basiry D. Kommedal R. Kaster K. (2025). Effect of subinhibitory concentrations on the spreading of the ampicillin resistance gene blaCMY-2 in an activated sludge microcosm. *Environ. Technol.* 46 1612–1624. 10.1080/09593330.2024.2394719 39215485

[B3] Bergé C. Waksman G. Terradot L. (2017). Structural and molecular biology of Type IV secretion systems. *Curr. Top. Microbiol. Immunol.* 413 31–60. 10.1007/978-3-319-75241-9_2 29536354

[B4] Brown C. Maile-Moskowitz A. Lopatkin A. Xia K. Logan L. Davis B. (2024). Selection and horizontal gene transfer underlie microdiversity-level heterogeneity in resistance gene fate during wastewater treatment. *Nat. Commun.* 15:5412. 10.1038/s41467-024-49742-8 38926391 PMC11208604

[B5] Cai L. Sun J. Yao F. Yuan Y. Zeng M. Zhang Q. (2021). Antimicrobial resistance bacteria and genes detected in hospital sewage provide valuable information in predicting clinical antimicrobial resistance. *Sci. Total Environ.* 795:148815. 10.1016/j.scitotenv.2021.148815 34247085

[B6] Cassini A. Högberg L. Plachouras D. Quattrocchi A. Hoxha A. Simonsen G. (2019). Attributable deaths and disability-adjusted life-years caused by infections with antibiotic-resistant bacteria in the EU and the European Economic Area in 2015: A population-level modelling analysis. *Lancet Infect. Dis.* 19 56–66. 10.1016/S1473-3099(18)30605-4 30409683 PMC6300481

[B7] Chen L. Zhou H. Cai J. Zhang R. Chen G. (2009). Detection of plasmid-mediated IMP-1 metallo-beta-lactamase and quinolone resistance determinants in an ertapenem-resistant *Enterobacter cloacae* isolate. *J. Zhejiang Univ. Sci. B* 10 348–354. 10.1631/jzus.B0820302 19434761 PMC2676414

[B8] Chi X. Guo J. Zhou Y. Xiao T. Xu H. Lv T. (2020). Complete-genome sequencing and comparative genomic characterization of an IMP-4 producing Citrobacter freundii Isolate from patient with diarrhea. *Infect. Drug Resist.* 13 1057–1065. 10.2147/IDR.S244683 32341658 PMC7166059

[B9] Costa T. Harb L. Khara P. Zeng L. Hu B. Christie P. (2021). Type IV secretion systems: Advances in structure, function, and activation. *Mol. Microbiol.* 115 436–452. 10.1111/mmi.14670 33326642 PMC8026593

[B10] Element S. Moran R. Beattie E. Hall R. van Schaik W. Buckner M. (2023). Growth in a biofilm promotes conjugation of a bla NDM-1-bearing plasmid between *Klebsiella pneumoniae* strains. *mSphere* 8:e0017023. 10.1128/msphere.00170-23 37417759 PMC10449501

[B11] Elliott A. Huang J. Neve S. Zuegg J. Edwards I. Cain A. (2020). An amphipathic peptide with antibiotic activity against multidrug-resistant Gram-negative bacteria. *Nat. Commun.* 11:3184. 10.1038/s41467-020-16950-x 32576824 PMC7311426

[B12] Feng W. Zhou D. Wang Q. Luo W. Zhang D. Sun Q. (2016). Dissemination of IMP-4-encoding pIMP-HZ1-related plasmids among *Klebsiella pneumoniae* and *Pseudomonas aeruginosa* in a Chinese teaching hospital. *Sci. Rep.* 6:33419. 10.1038/srep33419 27641711 PMC5027574

[B13] Guo J. Qiu X. Xie Y. Hua Z. Wang Y. (2024). Regulation of intracellular process by two-component systems: Exploring the mechanism of plasmid-mediated conjugative transfer. *Water Res.* 259:121855. 10.1016/j.watres.2024.121855 38838482

[B14] Hassoun-Kheir N. Stabholz Y. Kreft J. de la Cruz R. Romalde J. L. Nesme J. (2020). Comparison of antibiotic-resistant bacteria and antibiotic resistance genes abundance in hospital and community wastewater: A systematic review. *Sci. Total Environ.* 743:140804. 10.1016/j.scitotenv.2020.140804 32758846

[B15] He K. Xue B. Yang X. Wang S. Li C. Zhang X. (2022). Low-concentration of trichloromethane and dichloroacetonitrile promote the plasmid-mediated horizontal transfer of antibiotic resistance genes. *J. Hazard Mater.* 425:128030. 10.1016/j.jhazmat.2021.128030 34986571

[B16] Holt K. Wertheim H. Zadoks R. Baker S. Whitehouse C. Dance D. (2015). Genomic analysis of diversity, population structure, virulence, and antimicrobial resistance in *Klebsiella pneumoniae*, an urgent threat to public health. *Proc. Natl. Acad. Sci. U S A.* 112 E3574–E3581. 10.1073/pnas.1501049112 26100894 PMC4500264

[B17] Ilangovan A. Connery S. Waksman G. (2015). Structural biology of the Gram-negative bacterial conjugation systems. *Trends Microbiol.* 23 301–310. 10.1016/j.tim.2015.02.012 25825348

[B18] Jalal N. Al-Ghamdi A. Momenah A. Ashgar S. Bantun F. Bahwerth F. (2023). Prevalence and antibiogram pattern of *Klebsiella pneumoniae* in a tertiary care hospital in Makkah, Saudi Arabia: An 11-year experience. *Antibiotics (Basel).* 12:164. 10.3390/antibiotics12010164 36671365 PMC9854758

[B19] Jeon J. Lee J. Lee J. Park K. Karim A. Lee C. (2015). Structural basis for carbapenem-hydrolyzing mechanisms of carbapenemases conferring antibiotic resistance. *Int. J. Mol. Sci.* 16 9654–9692. 10.3390/ijms16059654 25938965 PMC4463611

[B20] Katagiri M. Kuroda M. Sekizuka T. Nakada N. Ito Y. Otsuka M. (2021). Comprehensive genomic survey of antimicrobial-resistance bacteria in the sewage tank replacement with hospital relocation. *Infect. Drug Resist.* 14 5563–5574. 10.2147/IDR.S336418 34984011 PMC8709547

[B21] Kim D. Cha C. (2021). Antibiotic resistome from the One-Health perspective: Understanding and controlling antimicrobial resistance transmission. *Exp. Mol. Med.* 53 301–309. 10.1038/s12276-021-00569-z 33642573 PMC8080597

[B22] Kubota H. Suzuki Y. Okuno R. Uchitani Y. Ariyoshi T. Takemura N. (2019). IMP-68, a novel IMP-type Metallo-β-Lactamase in imipenem-susceptible *Klebsiella pneumoniae*. *mSphere* 4:e00736-19. 10.1128/mSphere.00736-19 31666316 PMC6821933

[B23] Lerminiaux N. Cameron A. (2019). Horizontal transfer of antibiotic resistance genes in clinical environments. *Can. J. Microbiol.* 65 34–44. 10.1139/cjm-2018-0275 30248271

[B24] Li R. Xie M. Liu L. Huang Y. Wu X. Wang Z. (2020). Characterisation of a cointegrate plasmid harbouring blaNDM-1 in a clinical *Salmonella* Lomita strain. *Int. J. Antimicrob. Agents* 55:105817. 10.1016/j.ijantimicag.2019.09.021 31600557

[B25] Li W. Zhang G. (2022). Detection and various environmental factors of antibiotic resistance gene horizontal transfer. *Environ. Res.* 212:113267. 10.1016/j.envres.2022.113267 35413299

[B26] Liu Z. Wang Z. Lu X. Peng K. Chen S. He S. (2021). Structural diversity, fitness cost, and stability of a BlaNDM-1-bearing cointegrate plasmid in *Klebsiella pneumoniae* and *Escherichia coli*. *Microorganisms* 9:2435. 10.3390/microorganisms9122435 34946035 PMC8708245

[B27] Martin R. Bachman M. (2018). Colonization, infection, and the accessory genome of *Klebsiella pneumoniae*. *Front. Cell. Infect. Microbiol.* 8:4. 10.3389/fcimb.2018.00004 29404282 PMC5786545

[B28] Miyakoshi M. Ohtsubo Y. Nagata Y. Tsuda M. (2020). Transcriptome analysis of zygotic induction during conjugative transfer of plasmid RP4. *Front. Microbiol.* 11:1125. 10.3389/fmicb.2020.01125 32625173 PMC7314908

[B29] Navon-Venezia S. Kondratyeva K. Carattoli A. (2017). *Klebsiella pneumoniae*: A major worldwide source and shuttle for antibiotic resistance. *FEMS Microbiol. Rev.* 41 252–275. 10.1093/femsre/fux013 28521338

[B30] Palm M. Fransson A. Hultén J. Búcaro Stenman K. Allouche A. Chiang O. (2022). The effect of heavy metals on conjugation efficiency of an F-Plasmid in *Escherichia coli*. *Antibiotics (Basel)* 11:1123. 10.3390/antibiotics11081123 36009992 PMC9404890

[B31] Qiu Z. Yu Y. Chen Z. Jin M. Yang D. Zhao Z. (2012). Nanoalumina promotes the horizontal transfer of multiresistance genes mediated by plasmids across genera. *Proc. Natl. Acad. Sci. U S A.* 109 4944–4949. 10.1073/pnas.1107254109 22411796 PMC3323979

[B32] Queenan A. Bush K. (2007). Carbapenemases: The versatile beta-lactamases. *Clin. Microbiol. Rev.* 20 440–458. 10.1128/CMR.00001-07 17630334 PMC1932750

[B33] San Millan A. (2018). Evolution of plasmid-mediated antibiotic resistance in the clinical context. *Trends Microbiol.* 26 978–985. 10.1016/j.tim.2018.06.007 30049587

[B34] Svebrant S. Spörndly R. Lindberg R. Olsen Sköldstam T. Larsson J. Öhagen P. (2021). On-site pilot testing of hospital wastewater ozonation to reduce pharmaceutical residues and antibiotic-resistant bacteria. *Antibiotics (Basel)* 10:684. 10.3390/antibiotics10060684 34201188 PMC8228021

[B35] Verlicchi P. Al Aukidy M. Zambello E. (2015). What have we learned from worldwide experiences on the management and treatment of hospital effluent? - An overview and a discussion on perspectives. *Sci. Total Environ.* 514 467–491. 10.1016/j.scitotenv.2015.02.020 25698384 PMC7112026

[B36] Waksman G. (2019). From conjugation to T4S systems in gram-negative bacteria: A mechanistic biology perspective. *EMBO Rep.* 20:e47012. 10.15252/embr.201847012 30602585 PMC6362355

[B37] Wang Y. Lu J. Mao L. Li J. Yuan Z. Bond P. (2019). Antiepileptic drug carbamazepine promotes horizontal transfer of plasmid-borne multi-antibiotic resistance genes within and across bacterial genera. *ISME J.* 13 509–522. 10.1038/s41396-018-0275-x 30291330 PMC6331567

[B38] Winterbourn C. (2008). Reconciling the chemistry and biology of reactive oxygen species. *Nat. Chem Biol.* 4 278–286. 10.1038/nchembio.85 18421291

[B39] Wu J. Zhou J. Liu D. Wu J. He R. Cheng Z. (2023). Phthalates promote dissemination of antibiotic resistance genes: An overlooked environmental risk. *Environ. Sci. Technol.* 57 6876–6887. 10.1021/acs.est.2c09491 37083356

[B40] Xu J. Lin W. Chen Y. He F. (2020). Characterization of an IMP-4-producing *Klebsiella pneumoniae* ST1873 strain recovered from an Infant with a bloodstream Infection in China. *Infect. Drug Resist.* 13 773–779. 10.2147/IDR.S247341 32210591 PMC7069566

[B41] Xu L. Zhou Z. Zhu L. Han Y. Lin Z. Feng W. (2020). Antibiotic resistance genes and microcystins in a drinking water treatment plant. *Environ. Pollut.* 258:113718. 10.1016/j.envpol.2019.113718 31838385

[B42] Yang B. Wang Z. Jia Y. Fang D. Li R. Liu Y. (2022). Paclitaxel and its derivative facilitate the transmission of plasmid-mediated antibiotic resistance genes through conjugative transfer. *Sci. Total Environ.* 810:152245. 10.1016/j.scitotenv.2021.152245 34896514

[B43] Yu X. Zhou Z. Shuai X. Lin Z. Liu Z. Zhou J. (2023). Microplastics exacerbate co-occurrence and horizontal transfer of antibiotic resistance genes. *J. Hazard Mater.* 451:131130. 10.1016/j.jhazmat.2023.131130 36878032

[B44] Yu Z. Wang Y. Lu J. Bond P. Guo J. (2021). Nonnutritive sweeteners can promote the dissemination of antibiotic resistance through conjugative gene transfer. *ISME J.* 15 2117–2130. 10.1038/s41396-021-00909-x 33589766 PMC8245538

[B45] Zhang H. Liu J. Wang L. Zhai Z. (2021). Glyphosate escalates horizontal transfer of conjugative plasmid harboring antibiotic resistance genes. *Bioengineered* 12 63–69. 10.1080/21655979.2020.1862995 33345705 PMC8806241

[B46] Zhang H. Song J. Zheng Z. Li T. Shi N. Han Y. (2023). Fungicide exposure accelerated horizontal transfer of antibiotic resistance genes via plasmid-mediated conjugation. *Water Res.* 233:119789. 10.1016/j.watres.2023.119789 36863279

[B47] Zhang K. Xin R. Zhao Z. Li W. Wang Y. Wang Q. (2021). Mobile genetic elements are the Major driver of High antibiotic resistance genes abundance in the Upper reaches of huaihe River Basin. *J. Hazard Mater.* 401:123271. 10.1016/j.jhazmat.2020.123271 32629348

[B48] Zhang Y. Wang Q. Yin Y. Chen H. Jin L. Gu B. (2018). Epidemiology of carbapenem-resistant *Enterobacteriaceae* infections: Report from the China CRE Network. *Antimicrob. Agents Chemother.* 62:e01882-17. 10.1128/AAC.01882-17 29203488 PMC5786810

[B49] Zhao K. Li C. Li F. (2024). Research progress on the origin, fate, impacts and harm of microplastics and antibiotic resistance genes in wastewater treatment plants. *Sci. Rep.* 14:9719. 10.1038/s41598-024-60458-z 38678134 PMC11055955

[B50] Zhou Z. Shuai X. Lin Z. Liu Y. Zhu L. Chen H. (2021). Prevalence of multi-resistant plasmids in hospital inhalable particulate matter (PM) and its impact on horizontal gene transfer. *Environ. Pollut.* 270:116296. 10.1016/j.envpol.2020.116296 33341549

[B51] Zhuang M. Achmon Y. Cao Y. Liang X. Chen L. Wang H. (2021). Distribution of antibiotic resistance genes in the environment. *Environ. Pollut.* 285:117402. 10.1016/j.envpol.2021.117402 34051569

