# Supplementary material for: Enhancement of blaIMP-carrying plasmid transfer in Klebsiella pneumoniae by hospital wastewater: a transcriptomic study
Source: Front Microbiol. 2025 Jul 28;16:1626123. doi: 10.3389/fmicb.2025.1626123 (PMC12336211; doi:10.3389/fmicb.2025.1626123)
Supplement: Supplementary file 2 [file Data_Sheet_2.docx]

**Supplementary Information**

**Text S1:** **Primer sequences and amplification condition for *bla*_IMP_**

Primer-F: 5’ - GGTGGTTGTTTTATTAAACCGTA - 3’

Primer-R: 5’ - TTTAACCGCCTGCTCTAATG - 3’

Reaction condition:

94 ℃ 5 min

94 ℃ 30 s

54 ℃ 30 s 31 cycles

72 ℃ 15 s

72 ℃ 6 min

**Text S2: Sequencing process, methods, and bioinformatics analysis of RNA-seq**

**1. RNA extraction, Library preparation, and Sequencing**

Total RNA was extracted using TRIzol® Reagent following the manufacturer's protocol. The RNA quality was assessed using a 5300 Bioanalyser (Agilent) and quantified with the ND-2000 (NanoDrop Technologies). Only high-quality RNA samples (OD260/280 = 1.8~2.2, OD260/230 ≥ 2.0, RIN ≥ 6.5, 28S:18S ≥ 1.0, > 1μg) were used for library construction. The mRNA libraries were prepared with the Illumina Stranded Total RNA Prep, Ligation with Ribo-Zero Plus kit. Sequencing was performed on an Illumina NovaSeq 6000 by Shanghai Majorbio Bio-pharm Biotechnology Co., Ltd. (Shanghai, China).

**2. Differentially Expressed Genes (DEGs) Identification**

The DESeq2^[1]^ package in R was used to identify significant DEGs in comparable groups (*P* < 0.05 and |Log2FC| > 1). The significantly DEGs were screened using thresholds of |LogFC| >1 and pdjust < 0.05. The Goatools tool was used for Gene Ontology (GO) term enrichment analysis, while the KOBAS tool was utilized for Kyoto Encyclopedia of Genes and Genomes (KEGG) functional enrichment analysis^[2]^. The significance level was set at *P* < 0.05, and the findings were displayed using a bubble chart.

**References**

[1] Love M I, Huber W, Anders S. Moderated estimation of fold change and dispersion for RNA-seq data with DESeq2[J]. Genome biology, 2014, 15(12): 550.

[2] Xie C, Mao X, Huang J, Ding Y, Wu J, Dong S, et al. KOBAS 2.0: a web server for annotation and identification of enriched pathways and diseases. Nucleic Acids Res 2011;39:W316-22.


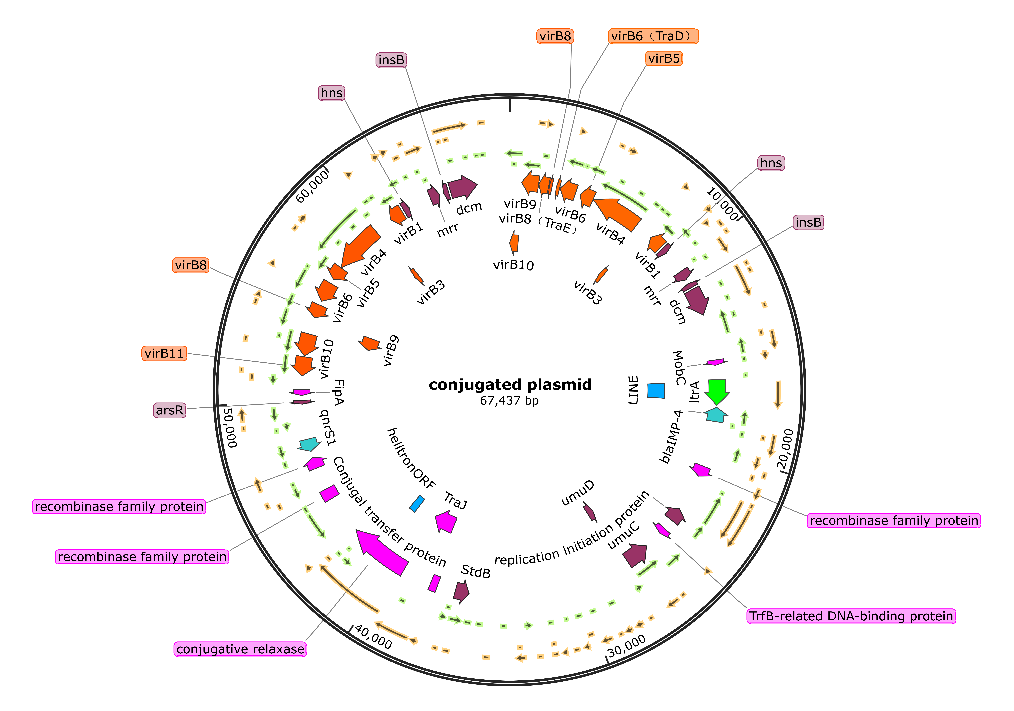


**Figure S1 Circle map of plasmid containing *bla*_IMP_**
